# Supplementary material for: A point-of-care microfluidic biochip for quantification of CD64 expression from whole blood for sepsis stratification
Source: Nat Commun. 2017 Jul 3;8:15949. doi: 10.1038/ncomms15949 (PMC5500847; doi:10.1038/ncomms15949)
Supplement: Supplementary Information [file ncomms15949-s1.pdf]

Type of file: PDF
Size of file: 0 KB
Title of file for HTML: Supplementary Information
Description: Supplementary figures, supplementary tables, supplementary notes and supplementary references.

Type of file: MP4
Size of file: 0 KB
Title of file for HTML: Supplementary Movie 1
Description: Recorded movie for the experimental visualization of beads-pillars interaction. Beads are interacting with the pillars after every 7th subsequent row having a pillars staggering ratio of 0.142 in the capture chamber.

Type of file: MP4
Size of file: 0 KB
Title of file for HTML: Supplementary Movie 2
Description: Recorded movie for the experimental visualization of beads-pillars interaction. Beads are interacting with the pillars after every 5th subsequent row having a pillars staggering ratio of 0.2 in the capture chamber.

Type of file: MP4
Size of file: 0 KB
Title of file for HTML: Supplementary Movie 3
Description: Recorded movie for the experimental visualization of beads-pillars interaction. Beads are interacting with the pillars after every 4th subsequent row having a pillars staggering ratio of 0.25 in the capture chamber.

Type of file: MP4
Size of file: 0 KB
Title of file for HTML: Supplementary Movie 4
Description: Recorded movie for the experimental visualization of beads-pillars interaction. Beads are interacting with the pillars after every 3rd subsequent row having a pillars staggering ratio of 0.33 in the capture chamber.

Type of file: PDF
Size of file: 0 KB
Title of file for HTML: Peer review file
Description:

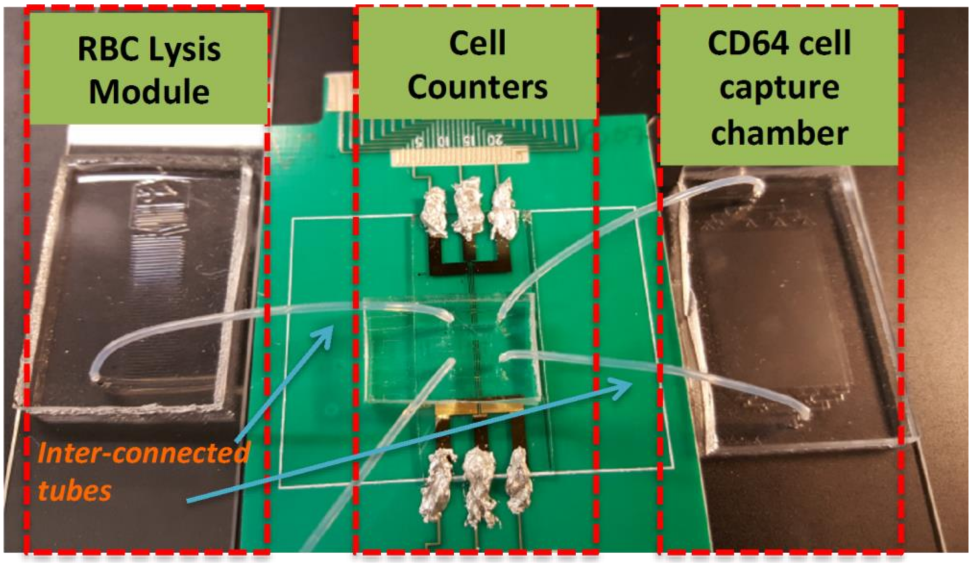

Supplementary Figure 1: The image shows the inter-connected modules of the sepsis biochip.

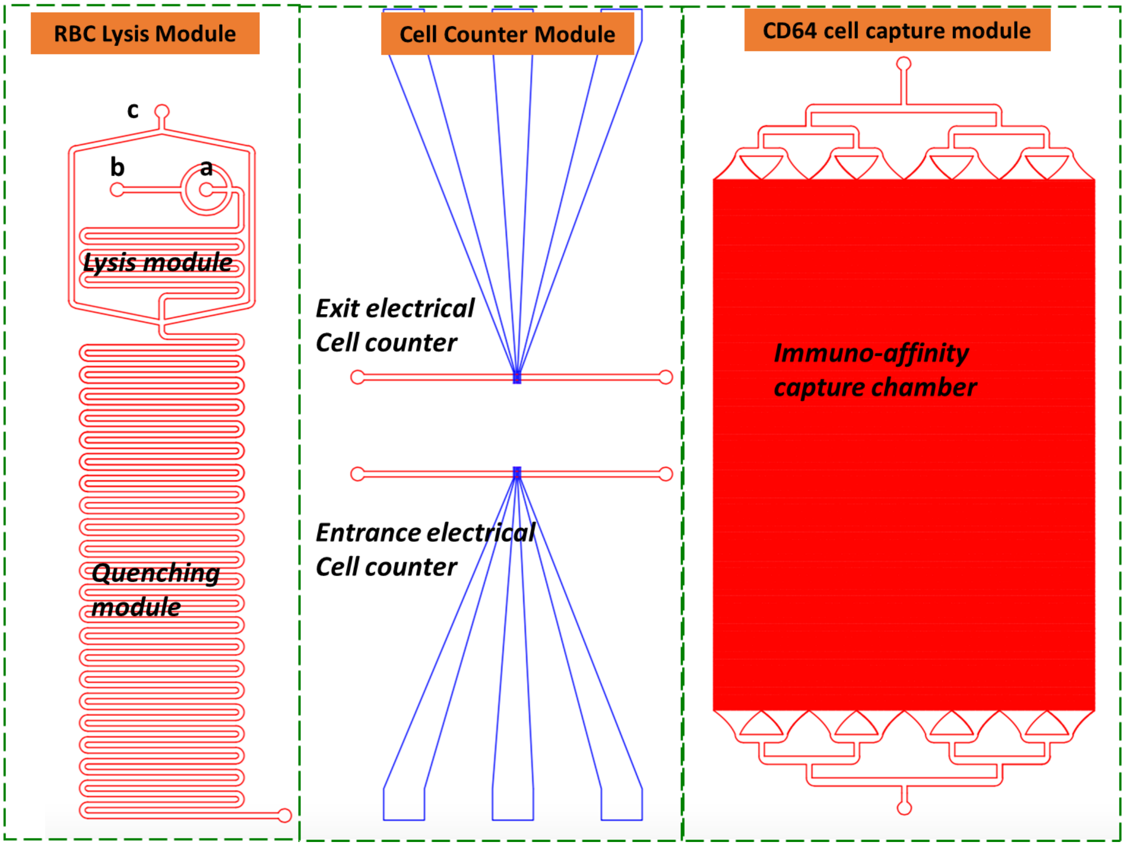

Supplementary Figure 2: The schematic overview of the sepsis biochip. 3 modules are shown, namely, erythrocyte (RBC) lysis module (left), cell counter module (middle) and CD64 cell capture module (right). Whole blood, lysing and quenching buffers are infused at a, b, c inlets respectively of lysis module. The three modules are interconnected with each other using PTFE tubing of ID 0.012 inch as shown in Supplementary Figure 1.

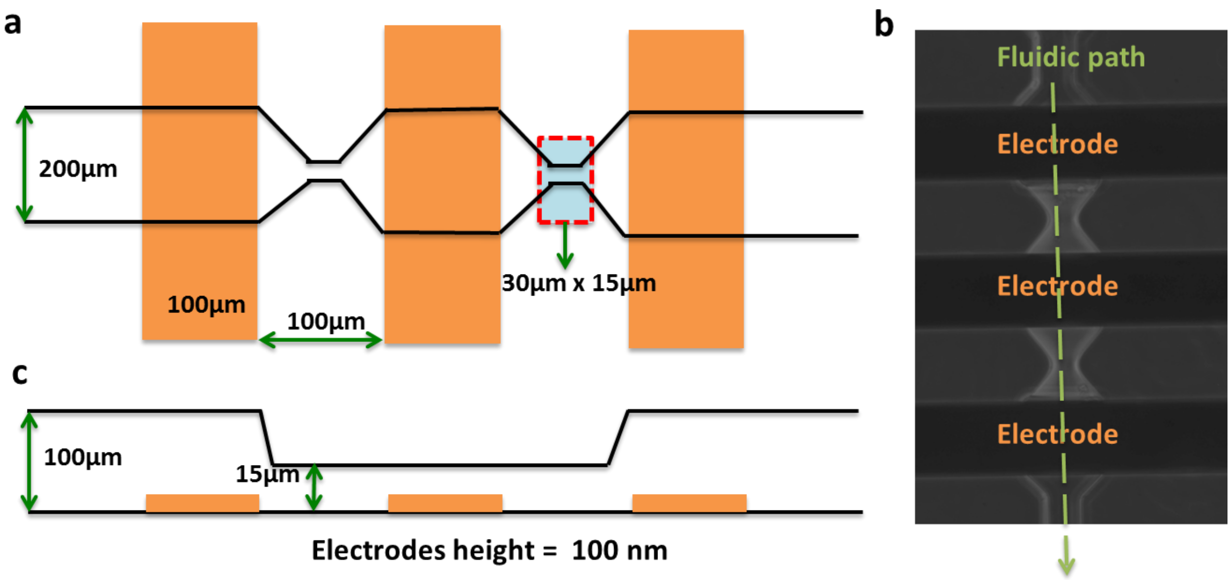

Supplementary Figure 3: Electrical counter design. (**a**) The top view schematic of the cell counters. The yellow bars represent electrodes of 100µm width and spacing in between them. The counting aperture is 30µm x 15µm. (**b**) The top-view image of a cell counter. Electrodes and the fluidic path are shown as labeled. (**c**) The cross-sectional schematic view of the cell counters. The electrodes are 100 nm in height (25 nm of titanium and 75 nm of platinum).

a

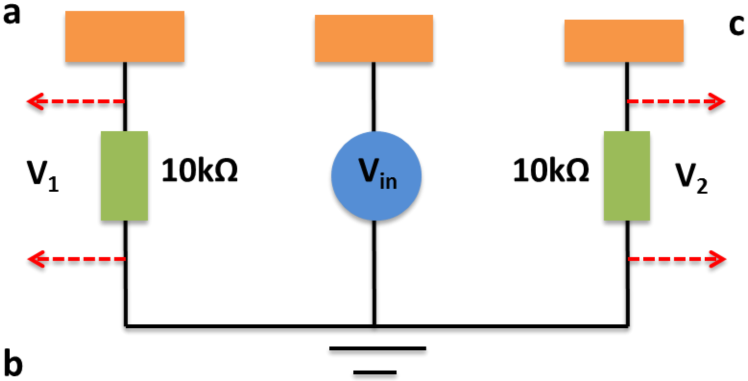

b

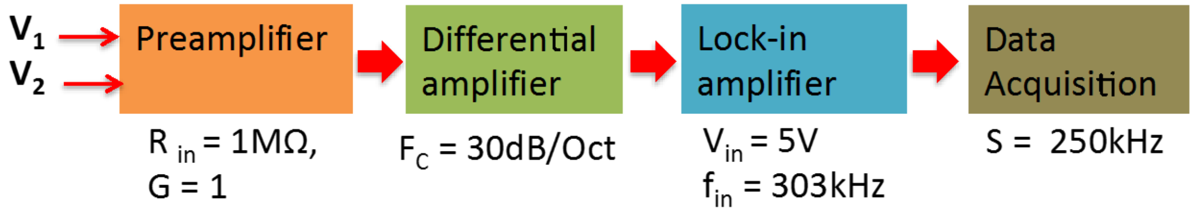

c

Filter Type	Cutoff frequency
High Pass	20 Hz
Band Stop	58, 62 Hz
Band Stop	118, 122 Hz
Low Pass	200 kHz

Supplementary Figure 4: The electronics scheme used in the setup. (**a**) Electrodes are fed with 5V and 303 kHz input signal using a built-in signal generator in lock-in amplifier. The output signal V1 and V2 are collected across 10k Ohms resistors. (**b**) The output signals are fed to the preamplifier with the input resistance of 1M Ohms and unity gain. The output signals are then fed to the differential amplifier to remove the common mode noise present in between the electrodes. The differential signal is then fed to lock-in amplifier, where it is filtered at the cut-off frequency of 30 dB per Oct. The filtered signal is sampled at 250 kHz using a DAQ card. (**c**) Digital filtering is performed at the sampled data using the following filters with their cutoff frequencies shown in order, high pass (20 Hz), band stop (58 - 62 Hz), band-stop (118 - 122 Hz) and low pass (200 kHz).

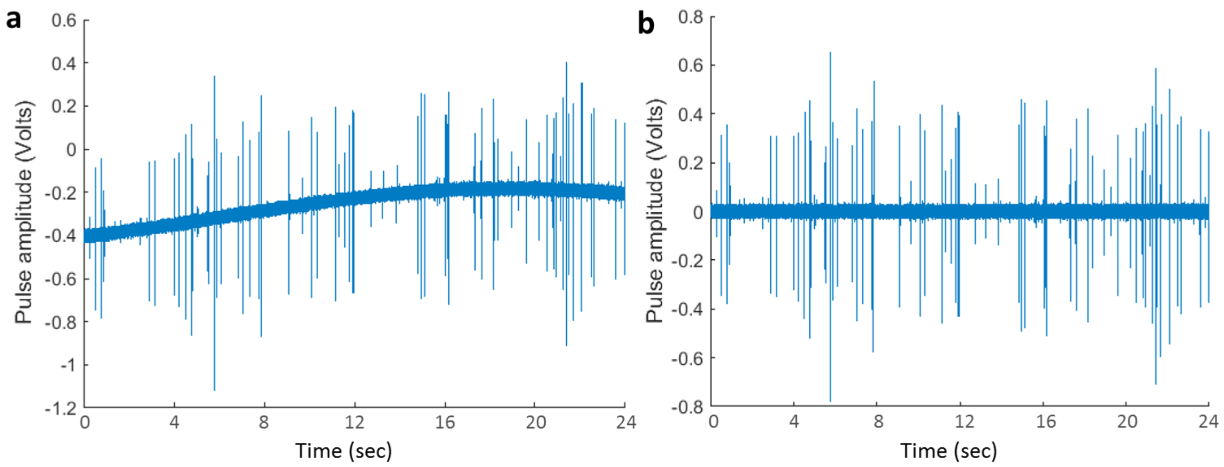

Supplementary Figure 5: The voltage pulses data as cell pass through the aperture. (**a**) Pulses data without any digital filtering shows the significant baseline drifts. (**b**) Pulses data after performing the digital filtering, the noise is removed and all the pulses are aligned to zero for accurate cell counting.

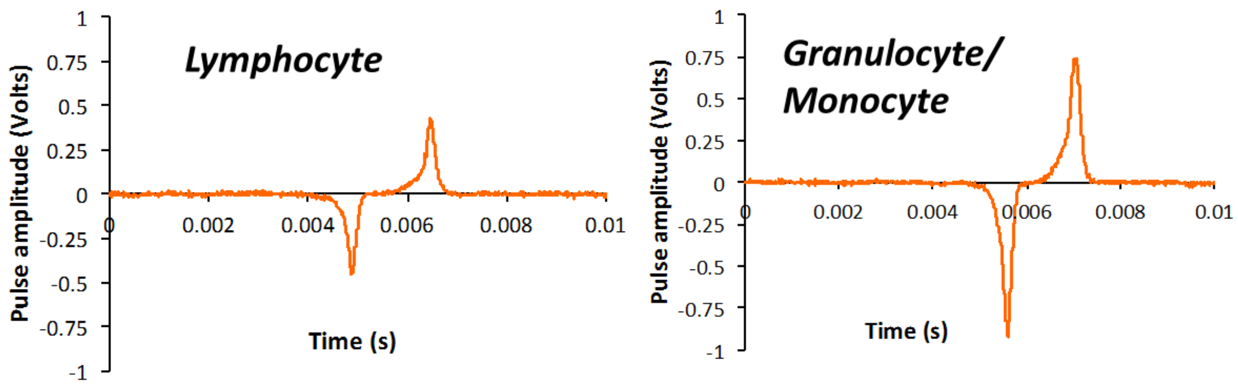

Supplementary Figure 6: The representative voltage pulses as cell pass through the counter. (**a**) Lymphocyte, and (**b**) Granulocyte/ Monocyte.

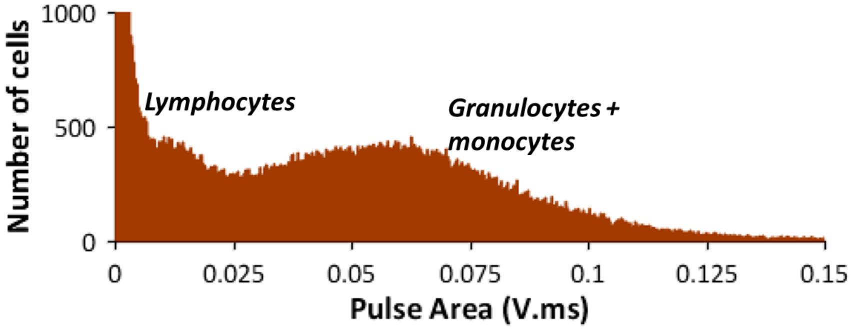

Supplementary Figure 7: The resulting pulse area histogram representing lymphocytes and granulocytes + monocytes as two populations.

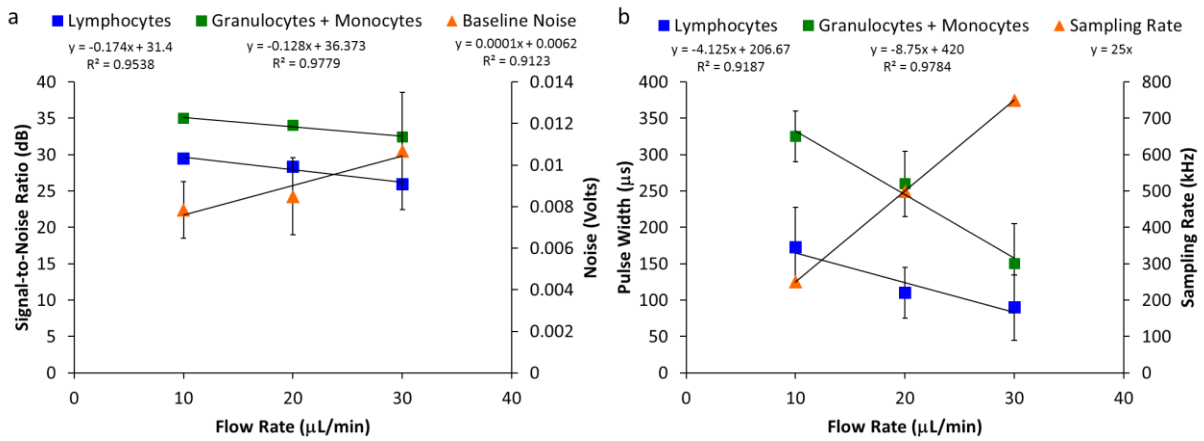

Supplementary Figure 8: The flow rate characterization with respect to the sensitivity of the electrical counter. (**a**) The plot shows the baseline noise (3 x standard deviation of noise) of the cell counting system also increases with increasing flow rates with corresponding decrease in the signal-to-noise ratio of the respective lymphocyte and granulocyte+ monocyte signals. (**b**) The pulse width at higher flow rates is smaller and have higher standard deviation making corresponding cell populations hard to distinct in the area histograms. And the sampling rate required to keep the same resolution (number of samples in a pulse) of the pulses, increase linearly with the increase in flow rates.

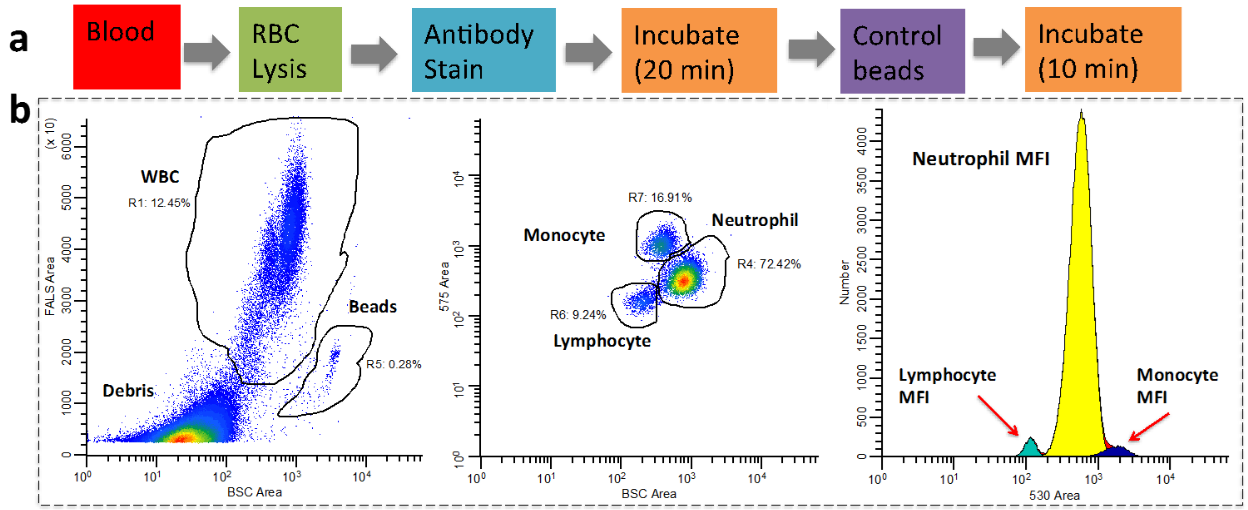

Supplementary Figure 9: CD64 expression quantification on a flow cytometer. (**a**) Stepwise blood processing protocol for CD64 expression quantification on a flow cytometer. (**b**) Flow cytometry analysis. The total leukocyte and control bead population can be identified and gated out on the forward-backscatter plot (left). The monocytes, neutrophils and lymphocytes can be easily differentiated and gated out on CD163 fluorescence vs. backscatter plot (middle). The fluorescence histogram (x-axis log scale) showing the fluorescence of CD64 fluorophores on neutrophils, lymphocytes and monocytes (right).

a

Parameter	SVM weight Coefficient
Gran + Mono	0.24
WBC	0.21
nCD64	0.20
Pulse	0.20
Lymphocytes	0.17
Temperature	0.16
Lactic acid	0.15
Blood Pressure	0.09
Respirations	0.05

b

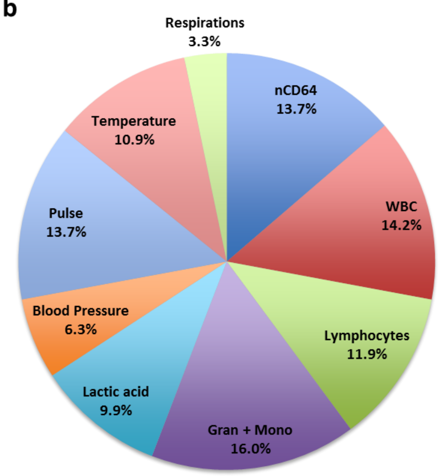

Supplementary Figure 10: (**a**) Table showing absolute values of the SVM weight vector coefficients of the parameters used in Fig. 2h for sepsis prediction. (**b**) The SVM coefficients are shown in the pie-chart form. Gran + Mono, WBC, and nCD64 are the 3 highest ranked parameters obtained for sepsis prediction.

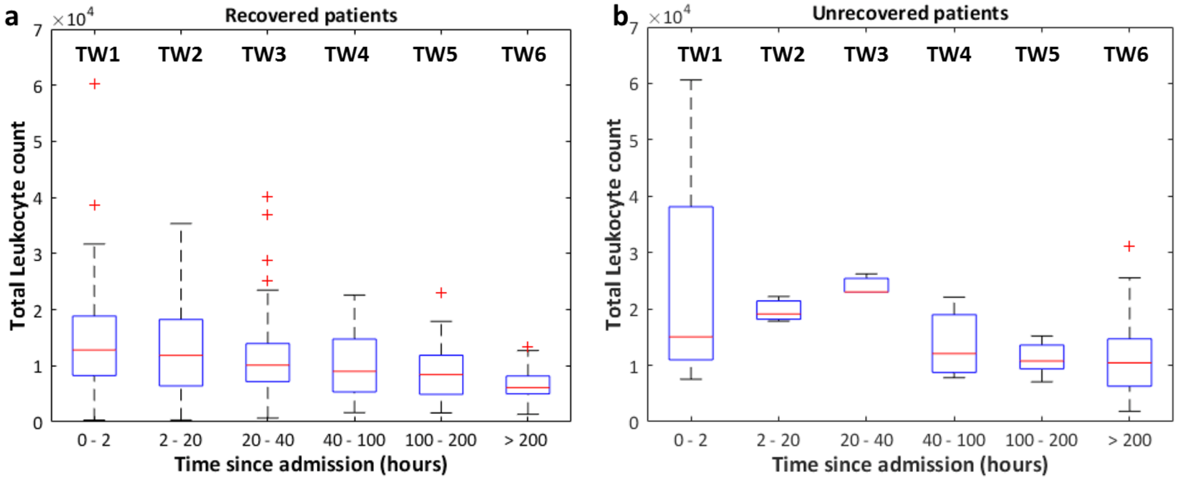

Supplementary Figure 11: (**a**) The box plots showing the total leukocytes counts from 316 blood samples collected from 68 patients (who recovered) at different times of their hospital stay. (**b**) The box plots showing the total leukocytes counts from 94 blood samples collected from 6 patients at different times of their hospital stay. Unfortunately, these patients didn't recover and died.

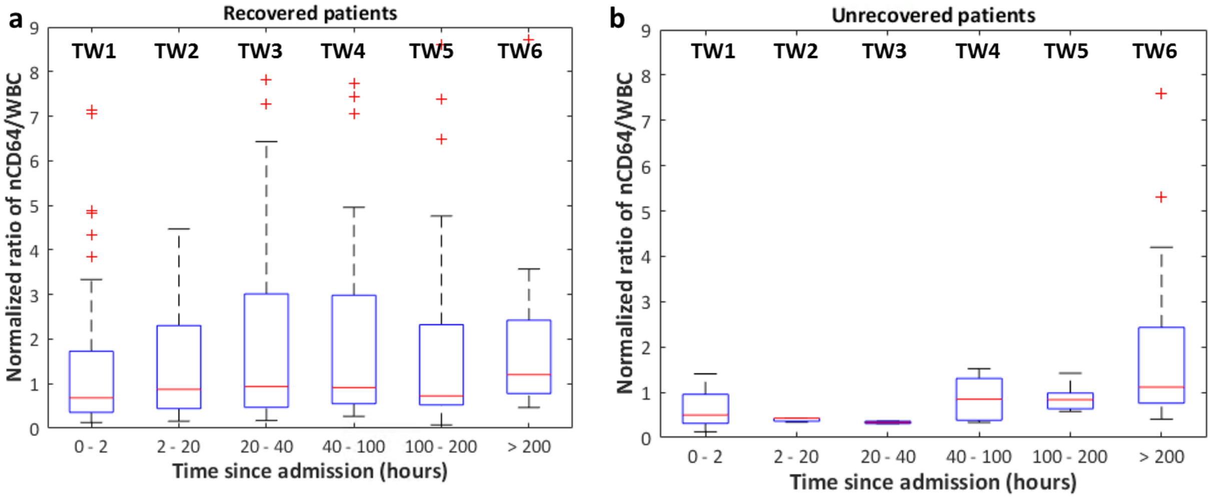

Supplementary Figure 12: (**a**) The box plots showing the normalized ratio of nCD64 to total leukocytes counts from 316 blood samples collected from 68 patients (who recovered) at different times of their hospital stay. (**b**) The box plots showing the normalized ratio of nCD64 to total leukocytes counts from 94 blood samples collected from 6 patients at different times of their hospital stay. Unfortunately, these patients didn’t recover.

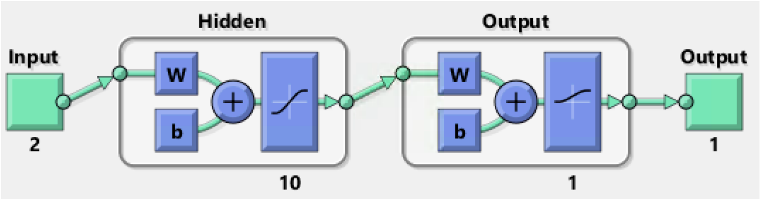

Supplementary Figure 13: The network diagram of a two-layer feed-forward artificial neural network with sigmoid hidden and softmax output neurons. It consists of 10 neurons in hidden and 1 neuron in output layers respectively. Input to the ANN model was total WBC count and nCD64 value at their respective time windows, and targets of the model are the patient's outcomes (recovered or died). The network was trained using scaled conjugate gradient backpropagation method.

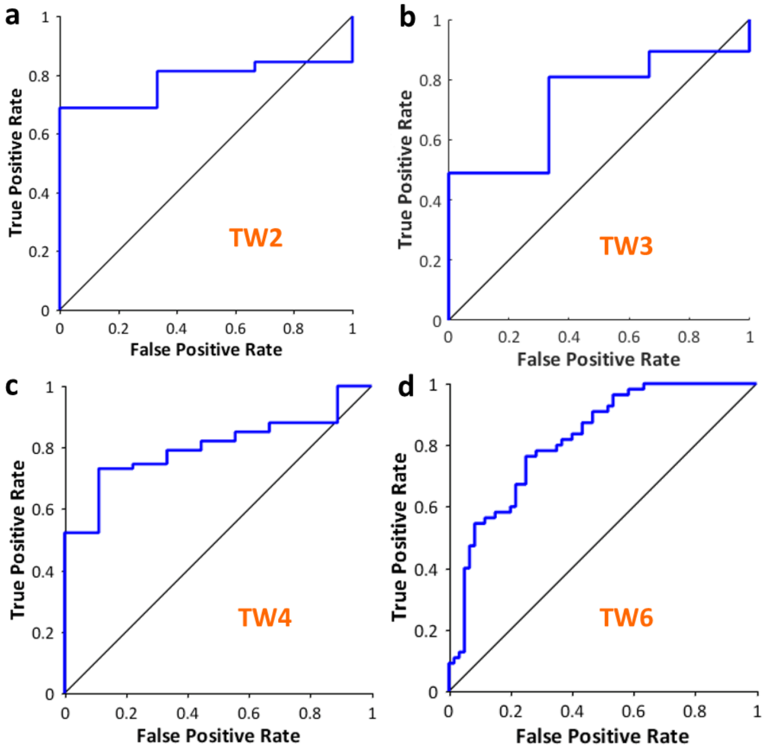

Supplementary Figure 14: The nCD64 and WBC counts are used for sepsis prognosis for all the six time windows using ANN model. ROC curves for (**a**) TW2 (AUC = 0.76), (**b**) TW3 (AUC = 0.68), (**c**) TW4 (AUC = 0.77), (**d**) TW6 (AUC = 0.78) for sepsis prognostication by predicting the recovery of the patients.

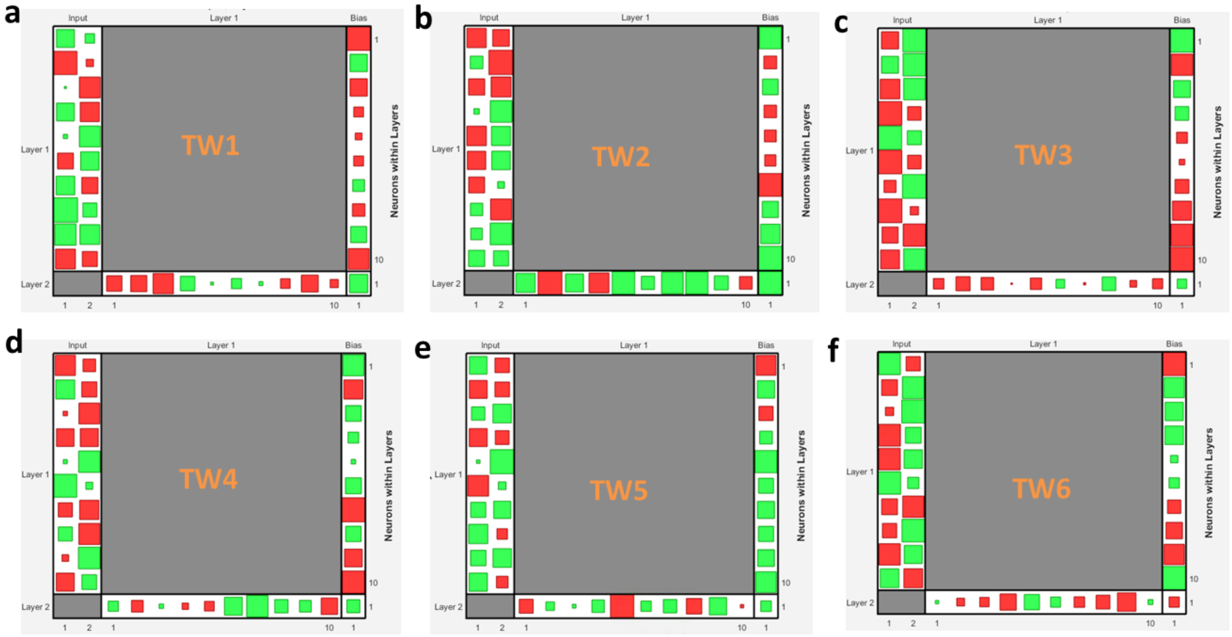

Supplementary Figure 15: Hinton diagram representing the weight and bias value of the input (hidden) and output layers of neurons used in our artificial neural network model. (**a-f**) shows the Hinton diagrams from TW1 to TW6 time windows.

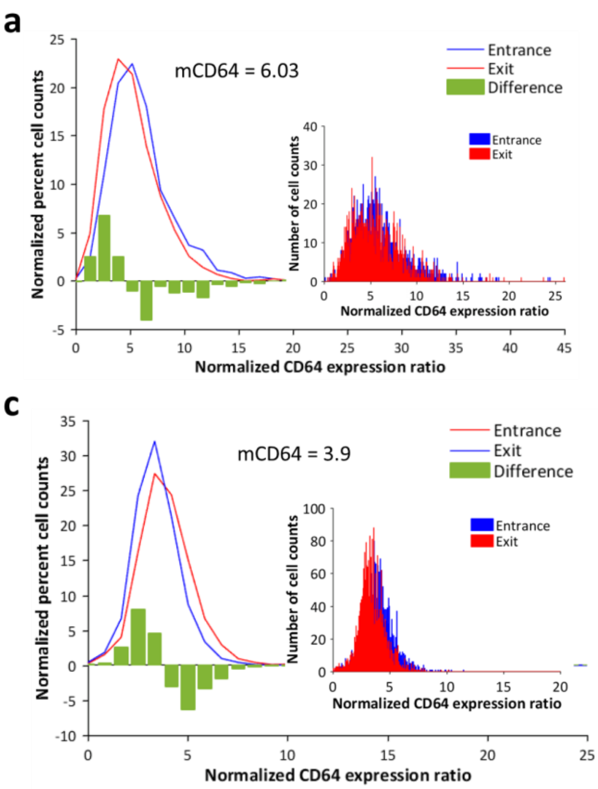

Supplementary Figure 16: Non-specific loss of granulocytes+ monocytes in the blocked chamber. (**a**) Non-specific capture of monocytes using Sample A (mCD64 = 6.03), with inset CD64 histograms of monocytes before capture (Blue) and after capture (Red) shows minimal capture. The red and blue curves show the exit and the entrance normalized percent monocyte count vs. mCD64 expression, with green bars representing difference of entrance minus exit cell counts. (**b**) Non-specific capture of neutrophils using Sample A (nCD64 = 2.43). (**c**) Non-specific capture of monocytes using Sample B (mCD64 = 3.9). (**d**) Non-specific capture of neutrophils using Sample B (nCD64 = 1.08).

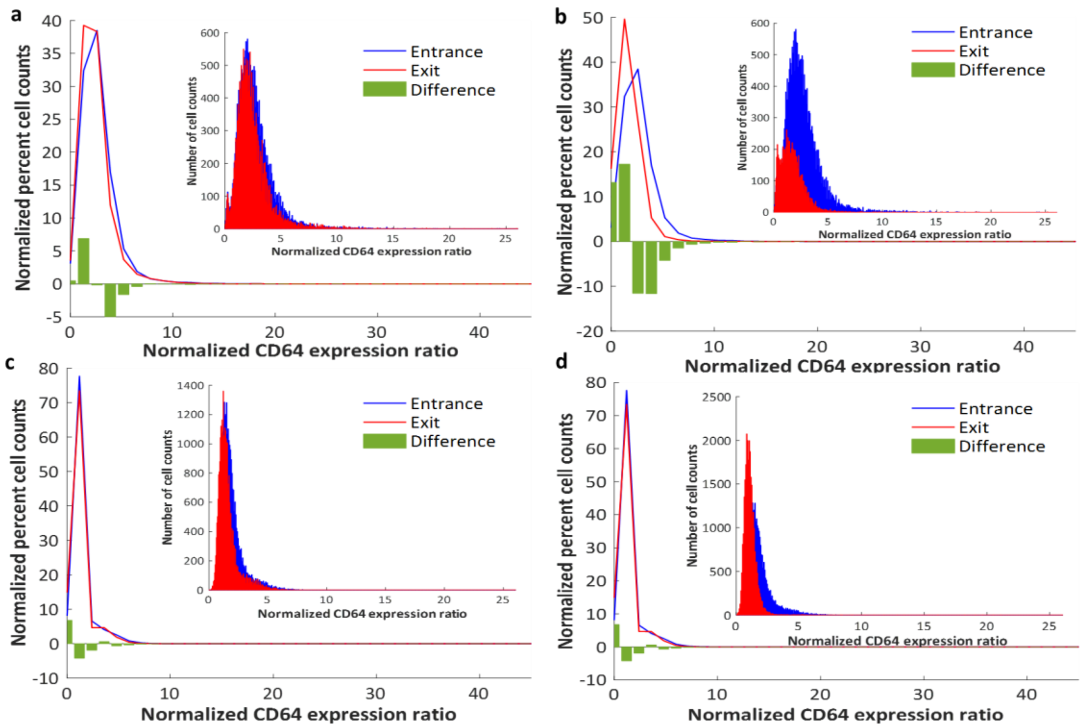

Supplementary Figure 17: (**a**) Non-specific capture of granulocytes + monocytes population using Sample A, with inset CD64 histograms of granulocytes + monocytes before capture (Blue) and after capture (Red) shows minimal capture. The red and blue curves show the exit and the entrance normalized percent granulocyte + monocyte count vs. normalized CD64 expression, with green bars representing difference of entrance minus exit cell counts. (**b**) CD64 expression based capture of granulocytes + monocytes using Sample A in an antibody chamber. (**c**) Non-specific capture of granulocytes + monocytes using Sample B in a blocked chamber. (**d**) CD64 expression based capture of granulocytes + monocytes using Sample B in an antibody chamber.

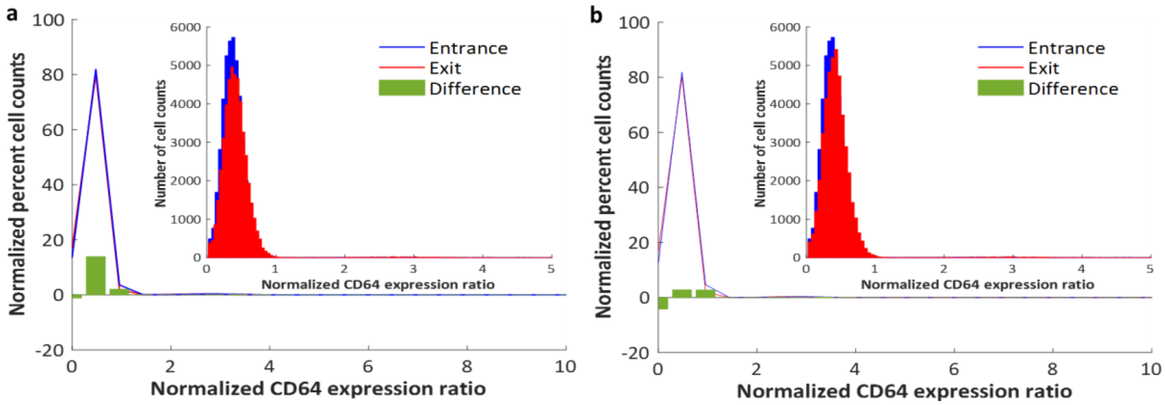

Supplementary Figure 18: (**a**) Non-specific capture of granulocytes using Sample C (nCD64 = 0.4), with inset CD64 histograms of granulocytes before capture (Blue) and after capture (Red) shows minimal capture. The red and blue curves show the exit and the entrance normalized percent granulocyte count vs. normalized CD64 expression. (**b**) CD64 expression based capture of granulocytes using Sample C in an antibody chamber. It shows the minimal capture.

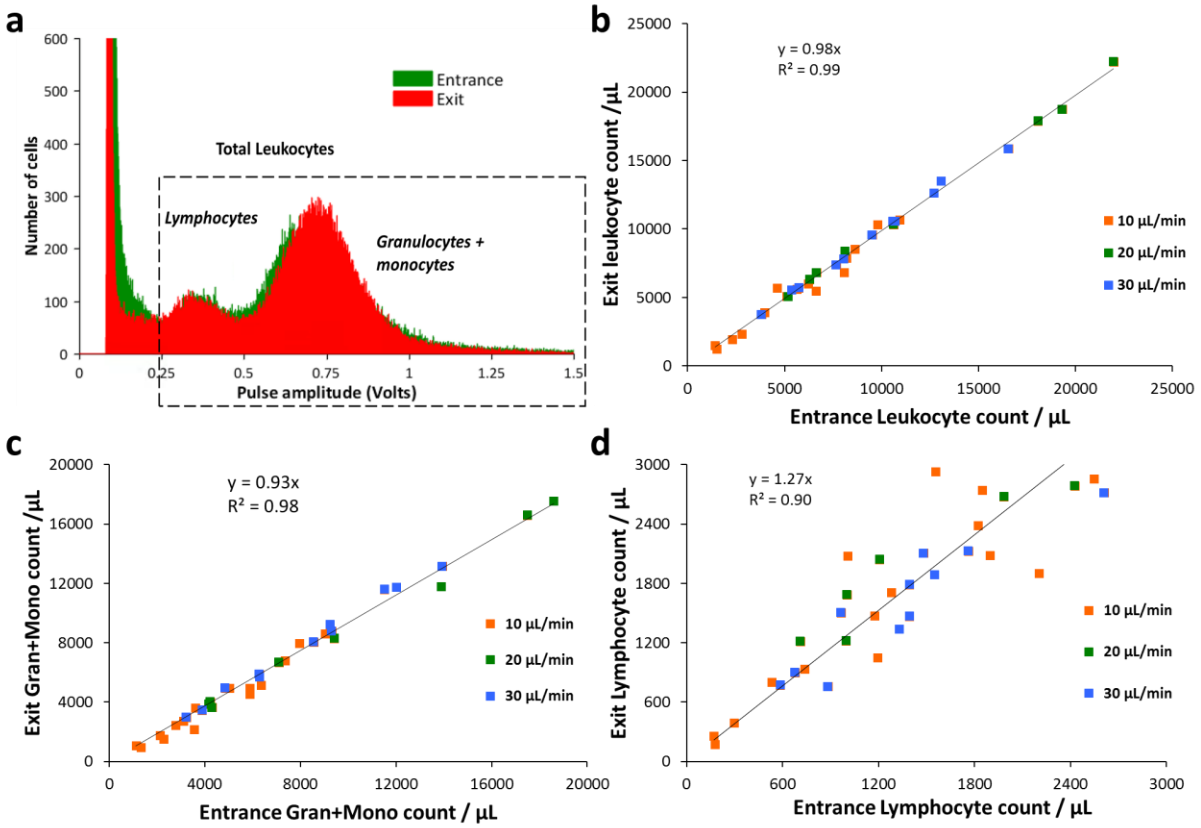

Supplementary Figure 19: Biochip investigation of the non-specific loss of granulocytes + monocytes in the blocked capture chamber. (**a**) The amplitude histogram of the cell pulses from the entrance counter (green) and exit counter (red). The recovery of the total leukocytes is 98% representing 2% loss of cells in the capture chamber. (**b**) The comparison of exit versus entrance total leukocyte counts. It shows the recovery of 98% with 2% loss of cells in chamber with (R^2 = 0.99). (**c**) The comparison of exit versus entrance total granulocyte + monocyte counts. It shows the recovery of 93% with 7% loss of cells in chamber with (R^2 = 0.98). (**d**) The comparison of exit versus entrance total lymphocyte counts. It shows the recovery of 127% with (R^2 = 0.90) due to cell permeabilization.

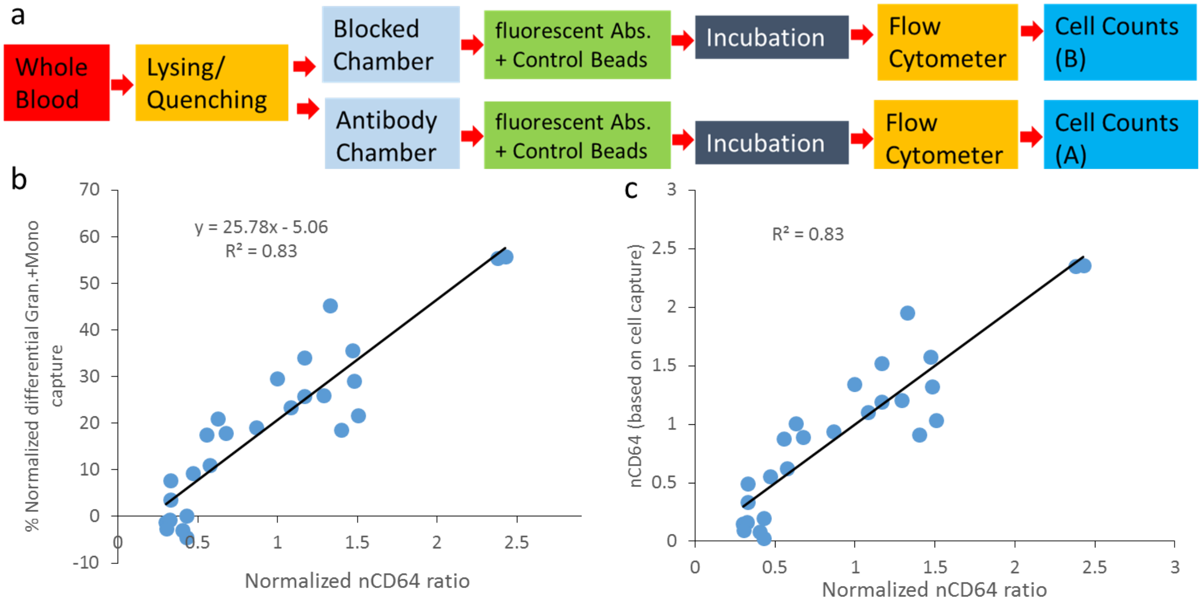

Supplementary Figure 20: Flow cytometry characterization of the nCD64 based cell capture. (**a**) Experimental protocol used, including of-chip lysing and quenching, and flowing cells through blocked and antibody chamber in parallel. The exit cells from chambers were labeled with fluorescent antibodies, then samples were incubated in dark for 20 min with subsequent flow through the cytometer. The number of cells counted from blocked (*B*) and antibody chambers (*A*). The percent cell capture is defined as $(A-B).B^{-1}$, normalized to the blocked chamber. (**b**) The average differential granulocyte + monocyte capture (from 3 replicates) vs. nCD64 ratio (n = 25 samples) shows a linear correlation of nCD64 with increase in the cell capture. (**c**) The plot shows the nCD64 based on cell capture (obtained using 3-fold cross-validation with 1000 random trials) vs. normalized nCD64 ratio.

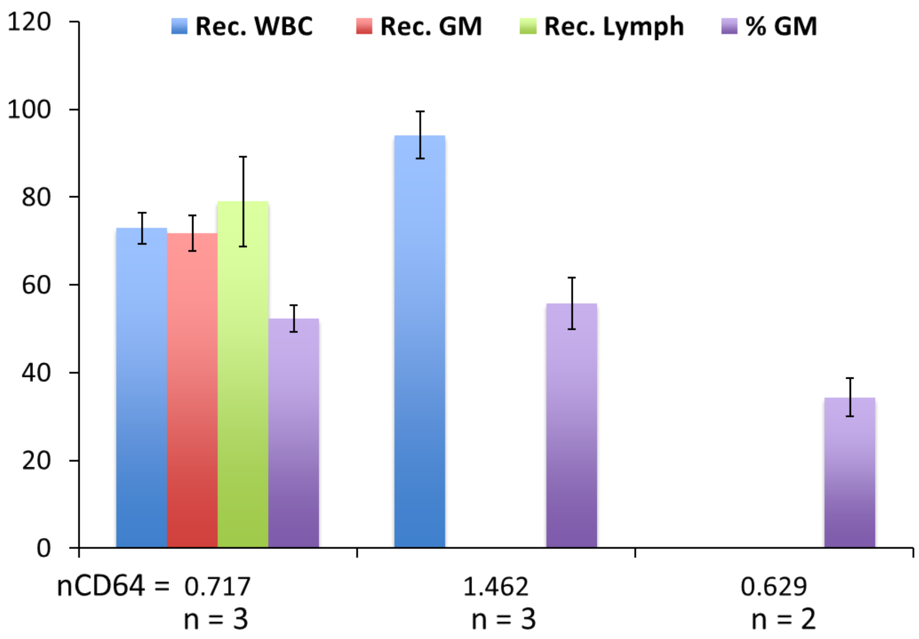

Supplementary Figure 21: Precision study

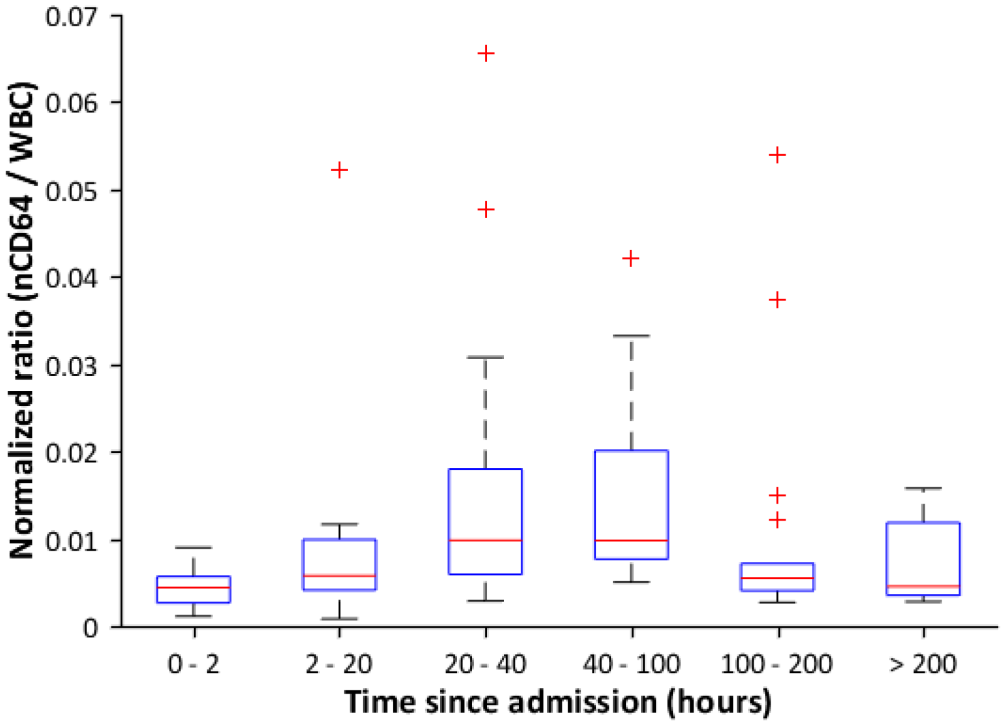

Supplementary Figure 22: The box plots showing the normalized ratio of nCD64 to WBC obtained from biochip from 91 blood samples collected from patients at different times of their hospital stay. All of these patients got recovered.

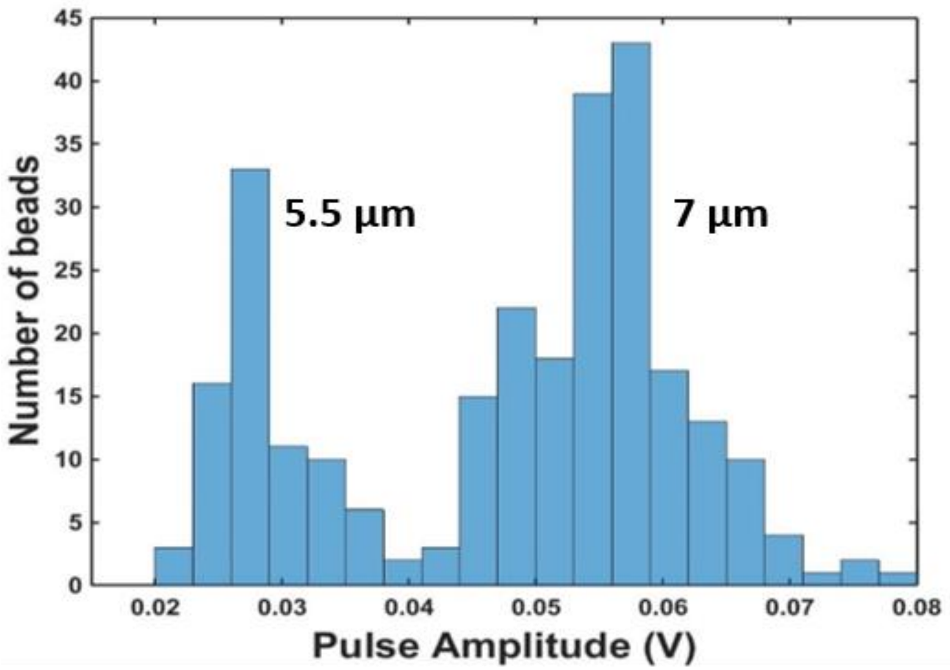

Supplementary Figure 23: The pulse amplitude histogram of the 5.5μm and 7μm beads flowing through the electrical counter. The counter is sensitive enough to differentiate in between these two beads populations.

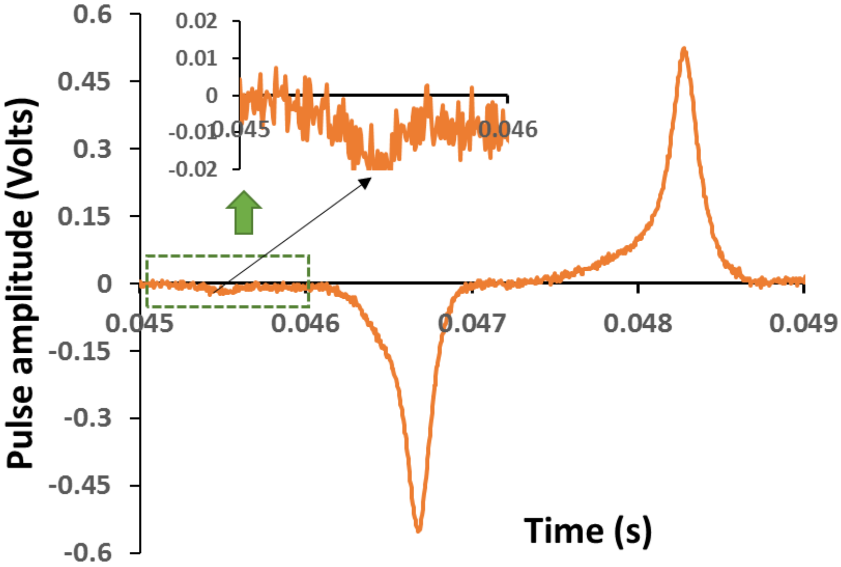

Supplementary Figure 24: The representative electrical voltage signal of a cell passing through the counter. The dotted rectangular region is zoomed-in to show the signal possibly related to the micro debris particles flowing through the counter channel.

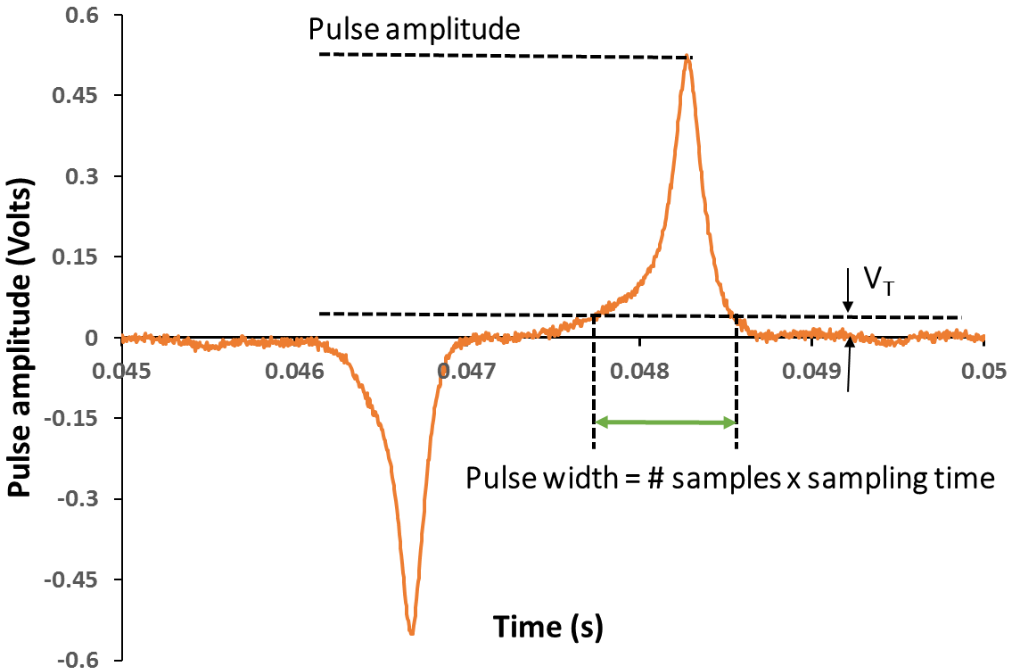

Supplementary Figure 25: The arrow in the dotted section shows the pulse width. Pulse width is calculated by measuring the number of samples in the dotted region and then multiplying it by sampling time. V_T is the cell detection threshold value.

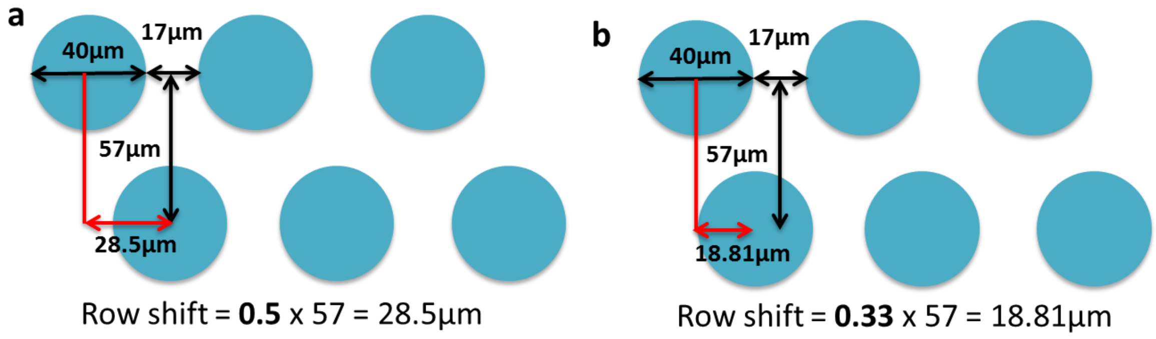

Supplementary Figure 26: The capture chamber pillars staggering dimensions. Width of each pillar is 40μm and row spacing in between pillars is 17μm. Each subsequent row is shifted using the following equation: Row shift = staggering ratio x (pillar-pillar) spacing. (**a**) For a staggering ratio of 0.5, rows are shifted by 28.5μm. (**b**) For a staggering ratio of 0.33, rows are shifted by 18.81μm.

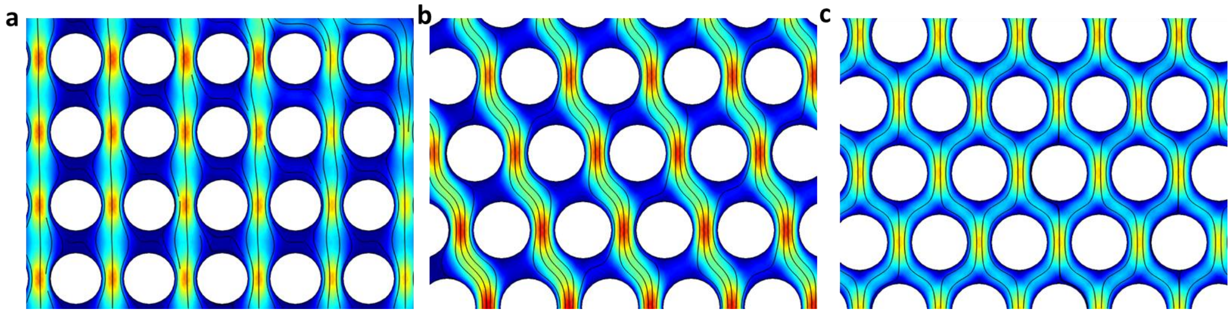

Supplementary Figure 27: COMSOL images of simulated capture chambers with different pillars staggering ratio along with flow streamlines around the pillars. (**a**) Staggering ratio of 0, if a particle is flown in middle streamline will never get a chance to interact with the pillar, (**b**) Staggering ratio of 0.33, streamlines shifts the position in between pillars, thereby making sure a particle will interact with the pillar more uniformly irrespective of the size. (**c**) Staggering ratio of 0.5, streamlines shifts the position in between pillars too.

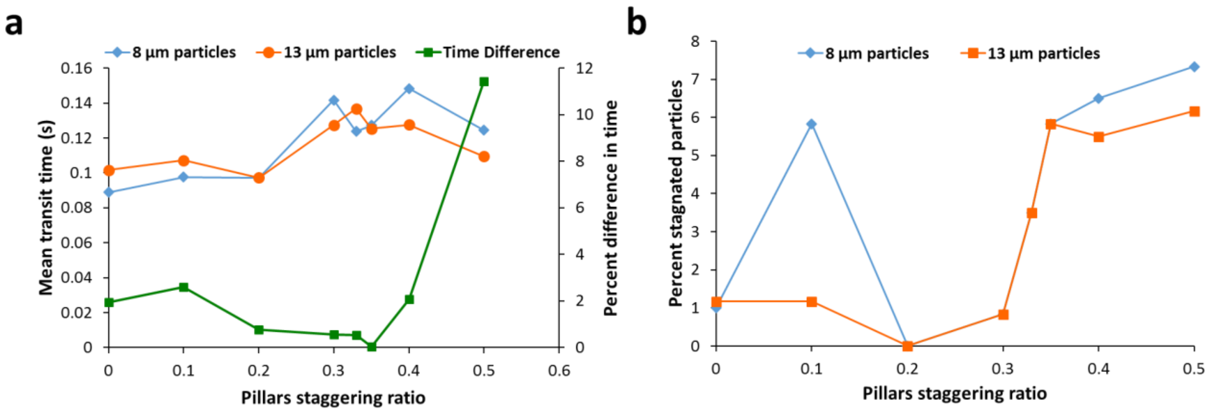

Supplementary Figure 28: Capture chamber design. (**a**) Simulated peak transit time and transit time difference (between 8μm and 13μm diameter particles) vs. pillars staggering ratio. (**b**) Pillars staggering ratio vs. simulated percent stagnated particles stuck at zero-velocity regions.

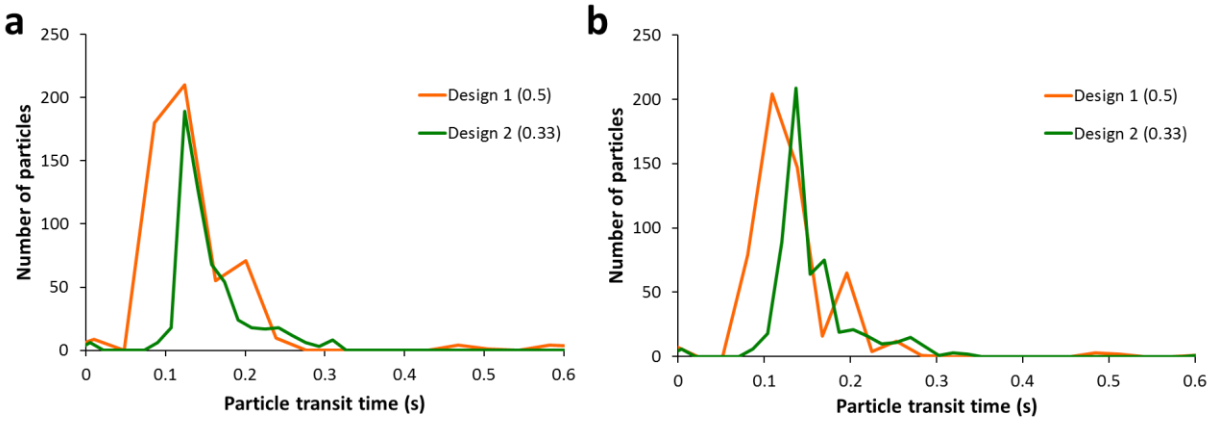

Supplementary Figure 29: Simulated comparison of the two chamber designs, with Design 1 and Design 2 having pillars staggering ratios of 0.5 and 0.33 respectively. (**a**) Particles (8μm diameter), and **(b)** Particles (13μm diameter), flowing through the Design 2 chamber have much uniform transit time profile indicating more uniform interactions with the pillars as compared to Design 1.

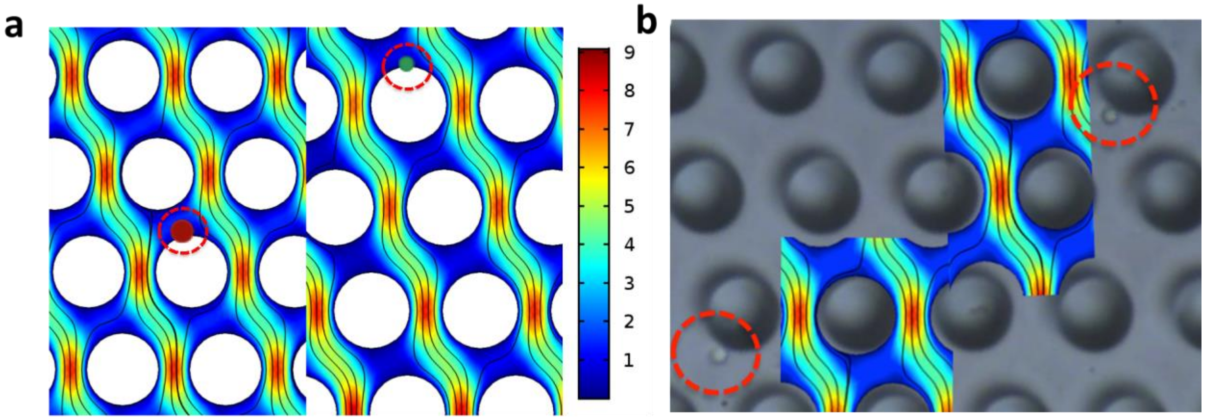

Supplementary Figure 30: (**a**) COMSOL images of simulated capture chambers with pillars staggering ratio = 0.33 with flow streamlines around the pillars. The red circles shows the potential stagnation (zero-velocity) regions where the particles are trapped. (Left: 13μm particles, Right 8μm particles). (**b**) The image of the real blocked capture chamber showing leukocytes stuck at zero-velocity regions with overlaid simulated streamlines.

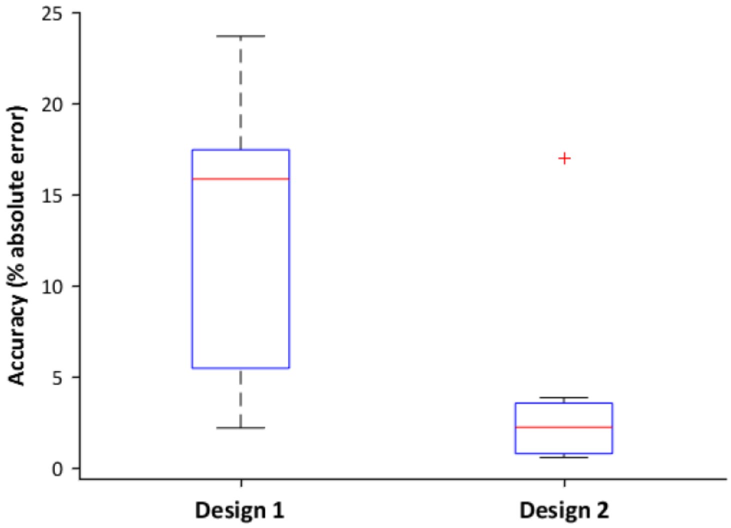

Supplementary Figure 31: Biochip investigation of the non-specific loss of granulocytes + monocytes in the blocked capture chamber using two different designs of capture chamber. The box plots representing accuracy as percent absolute difference in total leukocyte counts from entrance and exit counters while comparing Design 1 and 2 chambers using n = 8 blood samples respectively. The average percent difference for Design 1 and 2 chambers is 12.92% and 3.94% respectively.

Parameters	Dynamic Range	Average	Standard Deviation
**Leukocytes (cells/µL)**	530 – 38570	9925	5734
**Lymphocytes (cells/µL)**	110 – 2510	1144	643
**Granulocytes+ Monocytes (cells/µL)**	320 – 36580	8602	5525
**nCD64**	0.486 – 2.877	1.06	0.63
**mCD64**	1.21 – 14	4.11	1.92
**lCd64**	0.34 – 1.01	0.48	0.12

Supplementary Table 1: The dynamic range, average and standard deviation values of key parameters (leukocytes, lymphocytes, granulocytes + monocytes) of our targeted patient population.

Patient Characteristic		Non-septic Population (N=368)	Septic Population (N=76)
**Age (yr.), SD (yr.)**		61, 20	61, 19
**Gender (male, female)**		(56%, 44%)	(47%, 53%)
**Chronic Conditions**	Cancer	17%	20%
	Diabetes	19%	29%
	Kidney Disease	9%	9%
	COPD	10%	12%
	Asthma	1%	1%
**Infection (i.e. pneumonia, bronchitis, meningitis etc.)**		67%	89%
**Most common infections**	Pneumonia	23%	28%
	Cellulitis	19%	14%
	UTI	19%	41%
	GI infections	5%	11%
**Positive Blood Culture**		22%	38%
**SIRS positive**		64%	93%
**Infection + SIRS positive**		42%	79%

Supplementary Table 2: Patient characteristics including age, gender, chronic conditions and common infections with respect to septic and non-septic cohorts. SIRS positive is defined as if 2 or more out of 4 of the criteria parameters are valid.

	TW 1					TW 2					TW 3					TW 4					TW 5					TW 6				
	WI_1	WI_2	WO	I_b	O_b	WI_1	WI_2	WO	I_b	O_b	WI_1	WI_2	WO	I_b	O_b	WI_1	WI_2	WO	I_b	O_b	WI_1	WI_2	WO	I_b	O_b	WI_1	WI_2	WO	I_b	O_b
1	3.25	0.92	-2.44	-5.71	3.38	-3.53	-2.65	3.47	4.46	4.96	-2.26	4.03	-0.86	4.18	0.69	-4.12	-1.62	1.06	4.43	1.73	3.56	-2.44	-2.24	-4.55	1.31	3.91	-1.72	0.10	-4.60	-0.88
2	-5.45	-0.55	-2.68	3.06		1.52	-5.62	-5.35	-1.56		2.17	3.85	-1.52	-3.46		3.63	-2.33	-1.40	-3.69		-3.65	-2.50	0.85	3.44		-2.08	3.95	-0.52	3.39	
3	0.05	-4.41	-4.21	-3.06		-2.50	-3.78	3.08	2.33		-2.95	3.51	-1.20	2.17		-0.21	-4.37	0.21	2.49		2.06	4.00	0.16	-2.33		-0.58	4.40	-0.75	2.81	
4	3.24	-3.75	2.13	-0.99		0.29	4.42	-3.78	-1.48		-4.21	-1.40	-0.02	1.43		-3.17	-3.27	-0.42	1.18		-3.71	-2.20	1.78	1.86		-3.96	2.11	-2.22	2.87	
5	0.19	4.42	0.09	-0.48		-3.60	3.47	4.81	-1.35		4.11	1.50	-0.99	-0.92		0.18	4.59	-0.96	0.21		0.15	6.53	-6.27	5.58		-3.86	2.82	1.96	0.46	
6	-2.57	3.46	1.05	-1.02		-3.17	3.28	1.64	-1.20		-4.19	-1.41	0.59	-0.25		5.30	0.50	3.24	2.59		-4.89	0.68	1.24	2.59		4.17	1.06	0.86	0.91	
7	3.51	-2.69	0.16	1.41		-2.40	0.43	4.41	-4.90		-1.13	4.28	-0.04	-1.47		-2.03	-3.73	4.60	-5.78		2.62	3.37	2.08	3.33		-2.08	-3.77	-0.93	-1.52	
8	6.03	1.99	-0.96	-1.61		1.45	-4.16	4.61	2.48		-4.28	-0.54	1.43	-2.78		1.99	-4.46	1.81	2.38		4.25	-1.28	-2.74	2.66		-1.74	4.26	-1.60	-2.27	
9	4.60	3.96	-2.91	2.63		1.26	4.44	1.86	3.58		-1.98	-3.77	-0.36	-3.62		-0.46	4.71	1.39	-3.17		2.74	3.94	3.43	3.17		-3.80	2.73	-2.88	-3.45	
10	-4.03	-2.32	-0.69	-4.38		2.50	2.18	-1.52	5.52		-2.72	3.47	-0.80	-4.45		-3.27	2.22	-2.73	-4.96		3.79	-1.56	-0.13	4.84		2.93	-2.99	0.22	4.67	

Supplementary Table 3: The weight and bias values of all the neurons in input and output layers for all time windows.

Total Flow Rate (μLmin^{-1})	Blood Flow Rate (μLmin^{-1})	Lysis Flow Rate (μLmin^{-1})	Quenching Flow Rate (μLmin^{-1})	Lysis Region Volume (μL)	Quenching Region Volume (μL)	Lysing/ Quench Module Height (μm)	Chamber height (μm)	Experiment time (min)
10	0.518	6.218	3.264	0.685	4.405	90	60.1	23
20	1.036	12.435	6.528	1.370	8.810	180	84.84	13
30	1.554	18.653	9.793	2.055	13.214	270	103.92	10

Supplementary Table 4: Flow rate characterization with respect to the biosensor throughput. Table shows the changes in the biosensor design needed with increasing flow rates and corresponding decrease in the experimental assay time.

Supplementary Note 1: Theoretical Model of Expression Based Cell Capture:

A cell/ particle having a surface antigen density i.e. expression level of m_c flowing through the chamber, when gets in close proximity to the corresponding antibody/ receptors on the surface, with the receptor density of m_s. The antigen on the cell's surface will interact with the antibody receptors in the chamber for the bond formation. The bond formation process is stochastic in nature provided few molecules are in proximity of making a viable bond. The probability of bond formation can be estimated by using the probabilistic kinetic formulation of McQuarrie[1]. The probability of adhesion P_a defined as probability to get greater or equal to one antigen-antibody bond [2]. Furthermore, the probability of adhesion, P_a is directly proportional to the strength of the adhesive antigen-antibody bond. Considering the small density of antigen-antibody in a close proximity area, the steady-state P_a can be written as [3]:

$$P_a \cong m_s m_c K_a^0 A_c \, e^{\frac{-\delta f}{k_b T}}$$

Where A_c is area if interaction between cell and pillar, K_a^0 is the no-load association constant of antigen-antibody pair. f is the force per antigen-antibody pair, δ is the characteristic antigen-antibody bond length (1 Å), $k_b T$ is the Boltzmann constant[3]. Thus, from the equation, we can see that the by keeping the antibody density constant in the chamber, for a particular antigen-antibody interaction (fixed association/ dissociation constants), probability of adhesion increases linearly with the antigen density (expression level) on the cell's surface.

Supplementary Note 2: Flow Rate Characterization vs. Counter Sensitivity & Throughput.

Sensitivity of the impedance Sensing Module: In this study we varied the flow rate (10 - 30 $\mu Lmin^{-1}$) and investigated the noise levels in electrical signals, thereby impacting the sensitivity of the sensing module (electrical counter). We found that with increasing flow rates, the standard deviation of the baseline signal i.e. noise increases with increasing the flow rate. **Supplementary Figure 9a** shows that the increase in noise level with increase in flow rate and corresponding decrease in signal-to-noise ratios. Although the noise level is smallest at 10 μL/min, the increase in the noise at 30$\mu Lmin^{-1}$, is still workable as the pulse peak amplitudes will still be detected. Another consideration will be the sampling rate, as increasing the flow rates will require increasing the sampling rate as well. **Supplementary Figure 9b** shows the average pulse width of the lymphocytes and granulocytes + monocytes population, which decreases with increasing flow rates. Furthermore, the required sampling rate to keep same sampling resolution increases linearly. Currently, we have sampled the signal at 250 kHz, which provides enough resolution for pulse detection (each pulse comprised of 75-100 data points). To keep the same sampling resolution of the each electrical pulse at higher flow rates, the sampling rate should be increased linearly with the flow rate.

Increased Throughput vs. Flow Rate: In case of the throughput optimization, as the flow rate will increase, the experimental assay time will be reduced. Currently at 10$\mu Lmin^{-1}$, it takes around 23 min to finish the complete experiment. However, simple device modifications can easily result in much reduced experimental time of ~ 10 min. Some of the necessary modifications include:

1. In order to keep the lysing/ quenching times constant for optimized lysing process at higher flow rates, we need to increase the volume of the lysing/ quenching module, which can be easily done by increasing the height of the lysing and quenching channels.
2. Similarly, increasing the flow rate for higher throughput can change the shear stress in the chamber, thus producing different conditions for cell capture. To obtain the same shear stress, a simple modification is to increase the height of the chamber to keep the similar capture conditions for CD64 cell capture.

Supplementary Table 3 shows the flow rate characterization with respect to the biosensor throughput. It provides information on the necessary dimensional changes required in the biosensor design with increasing flow rates, which will also result in the corresponding decrease in the experimental assay time.

Supplementary Note 3: Required Blood Volume

We have used 10μL of blood sample to make the measurements. The volume of the blood (sample) required for the biomarker detection depends on the concentration of the biomarker in the blood. We believe 10 μL of blood have enough leukocytes (dynamic range of concentration = 530-38570 cells per μL) for the sensitive and accurate detection & counting of cells. For example, 10 μL volume of blood will definitely not be enough in the following cases where the biomarker concentration is very low:

1. Circulating Tumor Cell (CTC concentration: 1-10 cells per mL) measurements from whole blood
2. Other specific leukocyte biomarkers e.g. CD4+CD25+CD134+ T-cells (concentration: 1-5 cells per μL), potential biomarkers for latent TB detection.

Some of the recent studies have shown high variability in biomarker quantification from whole blood when collected from finger pricks[4]. However, we have used a drop of blood collected from venipuncture, which is easier to collect from the line port already installed in patient's arms. The need for using a small volume of blood (although its availability is not an issue in this setting) is still apparent in a PoC device, to reduce the number of reagents, time and associated costs for a test.

Furthermore, the major issue is the rapid sepsis stratification and the method of blood draw is not a limiting factor especially for the patients admitted to hospitals in particular ICUs with line ports installed.

Supplementary References

1. McQuarrie, D.A. Kinetics of small systems. *I. J Chem Phys* 38, 433–5, (1963).

2. Piper, J.W., Swerlick. R.A., Zhu. C. Determining force dependence of two-dimensional receptor–ligand binding affinity by centrifugation. *Biophys J* 74, 492–513, (1998).

3. Decuzzi, P., and Ferrari, M., The adhesive strength of non-spherical particles mediated by specific interactions Biomaterials 27, 5307–5314, (2006).

4. Bond, M.M., and Richards-Kortum, R.R., Drop-to-Drop Variation in the Cellular Components of Fingerprick Blood: Implications for Point-of-Care Diagnostic Development *Am J Clin Pathol* 144 (6), 885-894, (2015).
